# Transcriptomic Profiling of the Development of the Inflammatory Response in Human Monocytes *In Vitro*

**DOI:** 10.1371/journal.pone.0087680

**Published:** 2014-02-03

**Authors:** Paola Italiani, Emilia M. C. Mazza, Davide Lucchesi, Ingrid Cifola, Claudia Gemelli, Alexis Grande, Cristina Battaglia, Silvio Bicciato, Diana Boraschi

**Affiliations:** 1 Institute of Biomedical Technologies, National Research Council, Pisa/Segrate, Italy; 2 Department of Life Sciences, University of Modena and Reggio Emilia, Modena, Italy; 3 Department of Medical Biotechnology and Translational Medicine, University of Milano, Segrate, Italy; 4 Institute of Protein Biochemistry, National Research Council, Naples, Italy; Salute San Raffaele University School of Medicine, Italy

## Abstract

Monocytes/macrophages are key players in all phases of physiological and pathological inflammation. To understanding the regulation of macrophage functional differentiation during inflammation, we designed an *in vitro* model that recapitulates the different phases of the reaction (recruitment, initiation, development, and resolution), based on human primary blood monocytes exposed to sequential changes in microenvironmental conditions. All reaction phases were profiled by transcriptomic microarray analysis. Distinct clusters of genes were identified that are differentially regulated through the different phases of inflammation. The gene sets defined by GSEA analysis revealed that the inflammatory phase was enriched in inflammatory pathways, while the resolution phase comprised pathways related to metabolism and gene rearrangement. By comparing gene clusters differentially expressed in monocytes *vs.* M1 and *vs.* M2 macrophages extracted from an in-house created meta-database, it was shown that cells in the model resemble M1 during the inflammatory phase and M2 during resolution. The validation of inflammatory and transcriptional factors by qPCR and ELISA confirmed the transcriptomic profiles in the different phases of inflammation. The accurate description of the development of the human inflammatory reaction provided by this *in vitro* kinetic model can help in identifying regulatory mechanisms in physiological conditions and during pathological derangements.

## Introduction

In the healthy organism the innate immune system provides the first line of defence against external or internal danger signals, and functions by triggering a protective inflammatory reaction develops through different phases, from initiation to full inflammation and destruction of the initiating agent, followed by resolution, and re-establishment of tissue integrity with restoration of the physiological tissue functions. Briefly, when in a tissue a damage or an infection takes place, the innate immune system is activated, setting in motion a local inflammatory response that includes the recruitment of leukocytes from blood (first neutrophils and them monocytes) and the production of a series of pro-inflammatory mediators, such as TNF-α and IL-1β, by local immune cells (in particular the resident macrophages). NK and T cells can also enter the tissue in response to specific chemokines, and may influence the development of the inflammatory reaction by producing IFN-γ, a potent monocyte/macrophage inflammatory activator. An inflammatory reaction must be tightly controlled to avoid excessive collateral damages to host tissues, and the possible degeneration into pathological conditions (*e.g.*, chronic inflammatory or autoimmune diseases). Thus, a crucial commitment made in late inflammation is to convert the response from the cytocidal tissue-damaging mode to a tissue-repairing mode. Clearance of the initiating stimulus (*e.g.*, elimination of the infectious microorganisms) determines the cessation of the inflammatory stimulation and the concomitant activation of down-regulatory mechanisms (in which cytokines such as IL-10 are involved), leading to resolution of inflammation. Then, in the different microenvironment, innate immune cells produce a series of growth factors (including VEGF and TGF-β) thereby taking part in the final phase of tissue re-construction and re-establishment of homeostasis [1].

Monocytes/macrophages are key players in inflammatory host defense, both by the direct elimination of foreign agents and as organisers of the different phases of the inflammatory process [2]. Circulating monocytes enter tissues and become inflammatory macrophages upon tissue damage. Resident tissue macrophages have a role in tissue surveillance and homeostasis [3]. Both incoming inflammatory monocytes and resident macrophages can undergo different activation processes as a consequence of microenvironmental tissue-derived (damage) or cell-derived signals (microbes, activated lymphocytes) [4]–[6]. Two broad macrophage functional phenotypes have been proposed, mirroring the Th1/Th2 polarization. The classically activated macrophages (M1) develop in response to inflammatory factors like the Th1 cytokine IFN-γ, LPS and TNF-α, and mediate resistance against intracellular parasites and tumours [7], [8]. Alternative M2 macrophages are activated by Th2 cytokines, or FcγR binding in the presence of TLR agonists, or glucocorticoids and anti-inflammatory molecules (M2a, M2b, M2c respectively), and take part in parasite clearance, dampening of inflammation, tissue remodelling, and tumour promotion [9], [10].

Several *in vitro* and *in vivo* studies suggest that polarised M1 and M2 macrophages can to some extent switch from a phenotype to the other. A controversial issue is whether M1 and M2 macrophages consist of phenotypically distinct subpopulations that can serve different functions [11], [12], or the same cells can shift from one to another functional phenotype based on microenvironmental signals [13]. While in several pathological conditions the latter seems to be the case (*e.g.* obesity-induced insulin resistance [14], atherosclerotic lesions [15], cancer [16], endotoxin tolerance [17]), it is still unknown if M1 and M2 macrophages can undergo dynamic transitions between different functional states during a “physiological” inflammatory response. Gene expression profiling approaches have been used to cast light on monocyte-to-macrophage differentiation and polarization processes, on the recognition of molecular signatures in M1 and M2 macrophages, and on the understanding of the plasticity of macrophage activation [18], [19]. In the last sixty years, many studies on macrophage activation in inflammation relied on *in vivo* mouse models [20], and on *in vitro* models based on isolated murine primary cells (mainly peritoneal or bone marrow derived macrophages) and on immortalised monocytic cell lines (human or murine) [21]. More recently, data on human primary cells have also become available, obtained with human monocytes/macrophages *ex vivo* or *in vitro* (primary macrophages isolated from tissues, *in vitro* differentiated myeloid precursors, *in vitro* matured macrophages, peripheral blood monocytes) [18]. These studies investigated the activation of monocytes/macrophages, and provided information about the modes of type I *vs.* type II inflammatory activation *vs.* deactivation of macrophages in the human being. However, no information is available on the kinetic development of the macrophage inflammatory reaction and on the possibility that the same cell population could be first polarised towards an effector inflammatory program and subsequently re-polarized to the deactivation program. In this context, this study aims at providing such characterisation by setting up a reliable and representative model, based on human primary monocytes, that allows us to accurately describing the development and regulation of human macrophage functions during the entire course of the inflammatory reaction.

## Materials and Methods

### Monocyte isolation and culture

Human monocytes were obtained from discarded buffy coats of healthy blood donors (see the paragraph “Ethics statement”). Donors were aged 19–56 years (average 35.5 years), included females and males (10+2) and belonged to three different ethnic groups, in the attempt to capture at least partially the human heterogeneity. According to the Italian law, blood donors were clinically healthy, were screened and found negative for HIV, HBV and HCV, and were within the normal range for CBC (complete blood count), glycemia, cholesterol, triglycerides, transaminases, creatinine, and blood protein level. Monocytes were obtained by isolating PBMC on Ficoll-Paque PLUS gradients (GE Healthcare, Bio-Sciences AB, Uppsala, Sweden) and subsequent separation with Monocyte Isolation kit II (Miltenyi Biotec, Bergisch-Gladbach, Germany). Monocytes isolated by this technique encompassed about 80% CD14^++^CD16^−^ cells, 2–6% CD14^++^CD16^+^ cells, and 7–10% CD14^dim^CD16^+^ cells, thus fully reflecting the distribution of blood monocyte subpopulations [22]. Only preparations with >98% purity (determined by differential staining on cytocentrifuge smears) and viability (trypan blue dye exclusion) were used.

Monocytes were cultured at 5×10^6^ cells/well in 6-well culture plates (Costar®, Corning Inc., Corning, NY) in 2 ml of RPMI 1640+Glutamax-I Medium (GIBCO®, Life Technologies, Paisley, UK) supplemented with 50 µg/ml Gentamicin (GIBCO®) and 5% heat-inactivated human AB serum (Sigma-Aldrich Inc., St. Louis, MO) in moist air with 5% CO_2_. Monocytes were sequentially exposed to mixtures of stimuli (see Results): hrCCL2 (10 ng/ml), hrTNF-α (10 ng/ml), hrIFN-γ (25 ng/ml), hrIL-10 (20 ng/ml), hrTGF-β (10 ng/ml) (all from R&D Systems, Minneapolis, MN), LPS (5 ng/ml; from *E.coli* serotype 055:B5; Sigma-Aldrich). Cells were washed and fresh medium added at 2, 14 and 24 h. Viability at 48 h always exceeded 80%.

Fresh monocytes were taken as time 0. Cells were harvested in 700 µl of Qiazol (Qiagen, Hilden, Germany) at 2, 2.5, 3, 3.5, 4, 14, 24, and 48 h. Supernatants were collected at 4, 14, 24, and 48 h.

### RNA isolation and microarray hybridization

Total RNA was extracted from monocytes of 12 individual donors (3 for the “early” series: 0, 2.0–3.5 h; and 9 for the “late” series: 0, 4–48 h), using Qiagen miRNeasy kit (Qiagen), quantified spectrophotometrically (ND-1000, NanoDrop Technologies, Wilmington, DE), and checked for integrity by microcapillary electrophoresis (Agilent 2100 Bioanalyzer; Agilent Technologies, Palo Alto, CA). Samples were prepared starting from 0.1–1 µg total RNA, using the GeneChip® 3′ IVT Express kit or the GeneChip® One Cycle cDNA Synthesis kit (Affymetrix, Santa Clara, CA), with identical results. Biotinylated cRNAs (15 µg) were fragmented and hybridized for 16 h at 45°C onto GeneChip® HG-U133 Plus 2.0 Arrays (Affymetrix). After washing and staining, arrays were scanned with the GeneChip® Scanner 3000 7G (Affymetrix) and fluorescent images were acquired and analyzed using GCOS software (Affymetrix) to generate a total of 60 raw intensity files (CEL files).

### Data analysis

Analysis was performed in R using Bioconductor libraries and R statistical packages. Signals were converted to expression values by robust multi-array average procedure [23] and HG-U133 Plus 2.0 custom Chip Definition Files (CDF) based on GeneAnnot [24] (CDF Version 2.1.0, GeneCards Version 2.41, GeneAnnot Version 1.9). Intensity levels for a total of 18862 custom probe sets were background-adjusted and normalised using quantile normalisation, and log_2_ expression values calculated using median polish summarisation. Raw data are available at Gene Expression Omnibus (GEO) GSE47122.

Genes with statistically significant differential expression during time series were identified using the microarray Significant Profiles method coded in the R package *maSigPro* [25]. MaSigPro first applies a least-square technique to estimate the parameters of a general regression model for each gene (make.design function) and then uses the regression coefficients of the model to identify genes with statistically significant changes in their expression profiles (p.vector, T.fit and get.siggenes functions). Since the time-course was composed of 9 points, we computed a regression fit for each gene using a polynomial with a degree of 3 (cubic regression model) and selected those regression models with an associated corrected p value≤0.05. P values have been corrected for multiple comparisons using the false discovery rate procedure (FDR), *i.e.*, setting the parameter Q = 0.05 in the p.vector function. Once the statistically significant gene models were determined, the regression coefficients were used to identify genes showing statistically significant expression changes over time. To do this, a second model was constructed using only significant genes and applying a variable selection strategy based on stepwise regression. Specifically, we selected the backward stepwise regression and, at each iteration, retained those variables with a p value≤0.01 (*i.e.*, set the T.fit parameters at step method = backward and alpha = 0.01). Finally, we generated the list of significant genes by setting an additional selection criterion based on the R-squared value of the second regression model (*i.e.*, set the get.siggenes parameters rsq = 0.6 and vars = all). Results have been visualised clustering genes into k = 9 groups, using maSigPro k-mean clustering and default value for k.

### Analysis of publicly available gene expression data

Gene expression data of human primary monocytes and macrophages were retrieved from GEO repository (http://www.ncbi.nlm.nih.gov/geo), using as inclusion/exclusion criterion the type of microarray technology (*i.e.*, only data obtained with Affymetrix HG-U133 microarrays were considered). Twenty-four series comprising 474 samples were downloaded from GEO, and 303 samples (corresponding to monocytes and macrophages, and excluding dendritic cells) were selected and organised in a proprietary database using the software A-MADMAN [26] (Table S1). Samples were manually re-annotated and tagged based on the meta-information provided by GEO and by the original publications. Finally, the meta-database comprised 62 samples labelled as untreated monocytes, and 46 and 20 samples as M1 and M2 activated monocytes/macrophages, respectively (Table S2). Gene expression profiles were generated starting from CEL files using an approach inspired by the generation of custom CDF [27]. In custom CDF, probes matching the same transcript, but belonging to different probes sets, are aggregated into putative custom-probe sets, each one including only those probes with a unique and exclusive correspondence with a single transcript. Similarly, probes matching the same transcript but located at different coordinates on different type of arrays may be merged in custom-probe sets and arranged in a virtual platform grid. As for any other microarray geometry, this virtual grid may be used as a reference to create the virtual-CDF file, containing the probes, shared among the platforms of interest, and their coordinates on the virtual platform, and the virtual-CEL files containing the intensity data of the original CEL files properly re-mapped on the virtual grid. Once defined the virtual platform through the creation of its custom-CDF and transformed the CEL files into virtual-CEL files, raw data, originally obtained from different platforms, are homogeneous in terms of platform and can be pre-processed and normalised adopting standard approaches, as RMA or GCRMA. Here, expression values were generated from intensity signals using the combined HG-U133A/HG-U133Av2/HG-U133 Plus2.0 virtual-CDF file, the custom definition files for human GeneChips based on GeneAnnot, and the transformed virtual-CEL files. Intensity values for a total of 12167 meta-probesets were background-adjusted, normalised using quantile normalisation, and gene expression levels calculated using median polish summarisation (RMA algorithm) [23]. The expression matrix has been analysed with the Significance Analysis of Microarray method (SAM) [28], coded in the samrRpackage (http://cran.r-project.org/web/packages/samr/index.html), to identify differentially expressed genes in the comparisons between subsets of monocytes tagged as untreated, M1, and M2 (128 samples, see Table S2). Specifically, in the comparison between untreated monocytes and samples labelled as M1 (or as M2), we used the two-class procedure, estimated the percentage of false positive predictions with 1000 permutations, and selected those transcripts whose q-value (*i.e.*, False Discovery Rate, FDR) was equal to 0. This selection was further refined setting the lower limit for fold change induction (or reduction) to 5 and 8, when considering the comparison between untreated monocytes and samples M1 or untreated monocytes and samples M2, respectively.

### Over-representation analysis

Over-representation analysis was performed using the Gene Set Enrichment Analysis (GSEA) software [29] and gene sets from the Molecular Signatures Database (http://www.broadinstitute.org/gsea/msigdb/index.jsp). GSEA was applied on log2 expression data of the entire time course. The median expression profiles of the 9 groups of genes identified by maSigPro was used as continuous phenotype labels, and the Pearson's correlation as the metric to select gene sets with expression patterns resembling those encoded in the phenotype labels. As gene sets we used KEGG, Biocarta, and Reactome lists of the C2: curated gene sets collection. Finally, gene sets were defined as significantly enriched if the False Discovery Rate (FDR) was <5% when using Pearson as metric and 1,000 permutations of gene sets.

### Gene expression validation by qRT-PCR

cDNAs were reverse-transcribed from total RNA (100 ng) using High Capacity cDNA Archive Kit (Applied Biosystems, Foster City, CA). TaqMan qPCR assays were performed with an ABI PRISM 7900 sequence detection system (Applied Biosystems), using TaqMan Universal PCR Master Mix (Applied Biosystems) in 50 µl reaction volume. Primers and probes for *IL6*, *TNFA*, *IL7R*, *CD163*, *MMP9*, *MAFB*, *KLF4*, *PPARG*, *PPARD*, *CEBPA*, and *GAPDH* were supplied by Applied Biosystems as pre-made solutions. Each cDNA sample was run in triplicate and qRT-PCR reactions were carried out on six independent samples. Statistical analysis was performed using the (2^−ΔΔCt^) method [30]. Results are expressed as mean ± standard error (SEM) of relative quantity (RQ) of mRNA level variations *vs.* calibrator (fresh monocytes).

### Protein detection by ELISA

Production of IL-6 and chemokines, CXCL8 (IL-8) and CCL5 (RANTES), was measured on cell supernatants by ELISA (R&D Systems Minneapolis, MN), according to manufacturer's instructions. Each sample was assayed in duplicate.

### Statistical analysis

The qRT-PCR and ELISA results are expressed as mean values ± SEM. Differences between groups were analyzed using ANOVA and Fisher's test. A *P* value<.05 was considered statistically significant.

### Ethics statement

No ethical approval or informed consent is required by the Italian law for discarded blood products. In any case, the use of the blood samples from normal donors for the study of monocyte activation and polarization was included in a collaborative study with Prof. Paola Migliorini on monocyte activation in normal and autoimmune subjects, which was approved by the Ethical Committee of the University of Pisa S. Chiara Hospital (prot. AOUP 33998 of September 29, 2006), and which is still ongoing. All samples of human blood included in this study were from anonymous donors and all were donated by Prof. Migliorini.

## Results

### The *in vitro* monocyte-based model of inflammation

**Figure 1:**
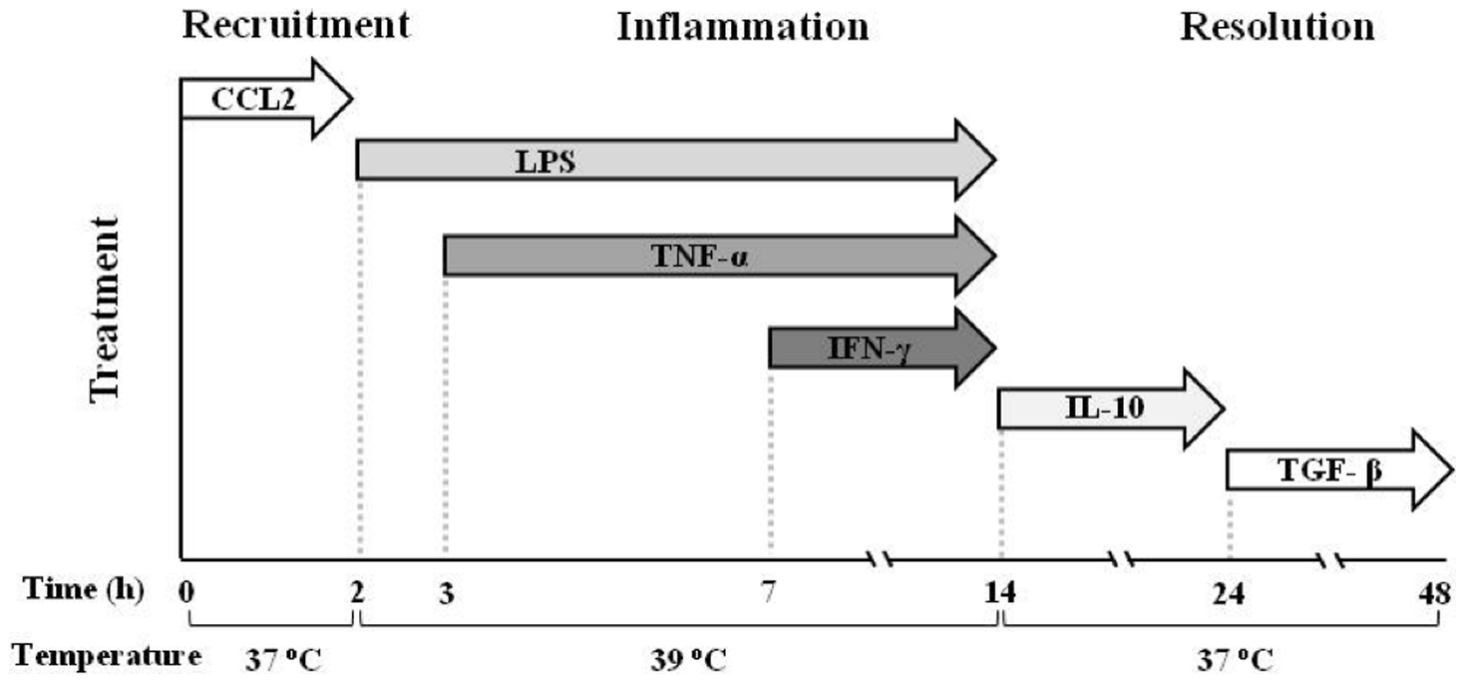
Graphic representation of the kinetic development of inflammation in the human monocyte-based *in vitro* model. Freshly isolated human blood monocytes were first exposed to the chemokine CCL2 for 2°C, then to LPS (from 2 h), TNF-α (from 3 h), and IFN-γ (from 7 h) at 39°C. At 14 h the inflammatory stimuli were washed off, the temperature brought back to 37°C, and fresh medium containing IL-10 added. At 24 h monocytes were exposed to TGF-β until the end of the experiment.

Blood monocytes from 12 individual healthy donors were exposed to a sequence of culture conditions mimicking the evolving microenvironment during an inflammatory reaction (Figure 1). Monocytes were initially exposed to CCL2 at 37°C, to represent recruitment to the site of inflammation, then to LPS and, sequentially, to TNF-α and IFN-γ at 39°C, to mimic the encounter with infectious agents and the inflammatory microenvironment (tissue reaction and influx of Th1 cells). At 14 h, culture conditions were changed (37°C and medium containing IL-10 first and subsequently TGF-β) to reproduce activation of anti-inflammatory mechanisms and macrophage deactivation during resolution.

### Distinct gene signatures are identified during the inflammatory response

Transcriptomic analysis was performed on monocytes from each individual donor at five different stages of activation in comparison to control fresh monocytes (time 0): early inflammation (2–4 h), late inflammation (14 h) (both corresponding to M1 polarization); early and late resolution (24 and 48 h) (different stages of M2c polarization).

**Figure 2:**
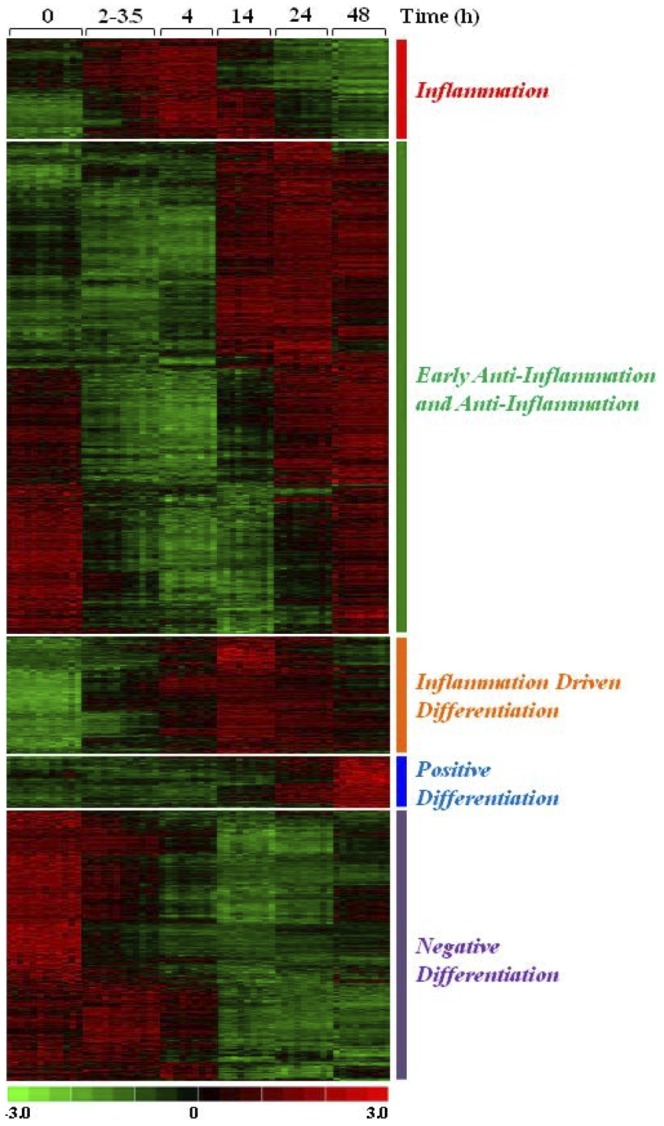
Differential gene expression during the inflammation phases. Heat-map showing the fold-expression levels of the genes that were identified by *maSigPro* as coherently downregulated (green) or upregulated (red) within the experimental set of 60 samples. Genes are organised into five major functional groups characterising the different phases of inflammation in this experimental setting: *Inflammation* (392), *Early Anti-Inflammation* and *Anti-Inflammation* (1871), *Inflammation Driven Differentiation* (457), *Positive Differentiation* (214) and *Negative Differentiation* (1061).

Genes showing statistically significant expression changes over time were identified by using the microarray Significant Profiles method (*maSigPro* R package) with parameters specified in (Figure S2). Results revealed profound changes in gene expression during the different phases of the inflammatory reaction, and the concomitant monocyte-to-macrophage differentiation. A total of 3995 genes (21.18% of the 18862 genes examined) resulted differentially expressed during the course of inflammation at a 95% confidence level (false discovery rate (FDR)≤0.05). Using k-means clustering method and *maSigPro* default parameters, significant genes were grouped in nine clusters showing distinct expression profiles during the inflammatory reaction (Figure S1). The nine clusters were merged into five major functional groups characterising the different phases of inflammation (Figure 2). The *Inflammation* functional group, encompassing clusters 1 and 2, is associated with the modulation of 392 transcripts. Of these, 218 are transiently upregulated during the first four hours of the inflammatory process, while 174 remain highly expressed during the late inflammation phase, and all decrease during the resolution phase. The *Early Anti-Inflammation*/*Anti-Inflammation* group, corresponding to clusters 3–5, contains 1871 genes, and includes genes that are downregulated in M1 polarised cells. Their median expression levels rapidly decrease upon stimulation with LPS/TNF-α, to eventually return to basal level in the resolution phase. Expression of the 457 genes in the *Inflammation Driven Differentiation* group (corresponding to cluster 6) rapidly increases upon inflammatory stimulation and remains elevated through the subsequent phases of the reaction. The *Positive Differentiation* group (cluster 7) includes 214 genes downregulated in fresh monocytes and during the early inflammation phases, but progressively upregulated during time with a transcriptional peak during late resolution. Conversely, the *Negative Differentiation* group (clusters 8 and 9) comprises a total of 1061 genes highly expressed in fresh monocytes and in early inflammation, and reduced during the subsequent phases. The list of all the 3995 differentially expressed genes, grouped in the nine clusters, is reported in the Table S3.

### Pathway analysis reveals relationship between activation and differentiation

Gene groups were subjected to GSEA for statistical associations between expression profiles of distinct groups and other gene signatures characteristic of various pathways or cellular processes described in KEGG, Biocarta and Reactome databases. We identified a total of 155, 358, 55, 149, and 66 pathways strongly associated with the expression profile of the five functional groups. The most representative gene sets associated with *Inflammation*, *Early Anti-Inflammation* and *Anti-Inflammation* are listed in Table 1. The majority of gene sets associated to *Inflammation* are pathways involved in classical inflammatory activation, and were not found associated to other groups (except *Inflammation Driven Differentiation*). The *Early Anti-Inflammation* and *Anti-Inflammation* clusters are enriched in pathways associated to metabolism and regulation of gene expression. The *Inflammation Driven Differentiation* group is associated to signalling cascades common to both inflammatory and anti-inflammatory phases, while pathways enriched in the *Positive Differentiation* and *Negative Differentiation* groups are similar to those found in the anti-inflammatory phase.

**Table 1:** Most representative gene sets associated with the *Inflammation*, *Early Anti-Inflammation* and *Anti-Inflammation* functional groups.

Functional groups	FDR q-val
***Inflammation***	
BIOCARTA_NFKB_PATHWAY	0.003
BIOCARTA_IL-1R	0.010
BIOCARTA_IL-10_PATHWAY	0.013
BIOCARTA_INFLAM_PATHWAY	0.021
BIOCARTA_CD40_PATHWAY	0.033
BIOCARTA_CYTOKINE_PATHWAY	0.044
KEGG_MAPK_SIGNLING	0.000
KEGG_CYTOKINE_CYTOKINE_RECEPTOR_SIGNALING	0.000
KEGG_NOD_LIKE_RECEPTOR_SIGNALING	0.000
KEGG_ECM_RECEPTOR_INTERACTION	0.001
KEGG_CELL_ADHESION_MOLECULES_CAMS	0.005
KEGG_PATHWAY_IN_CANCER	0.008
KEGG_JAK_STAT_SIGNALING	0.011
KEGG_NOTCH_SIGNALING	0.015
KEGG_TOLL_LIKE_RECEPTOR_SIGNALING	0.037
REACTOME_CHEMOKINE_RECEPTORS_BIND_CHEMOKINES	0.000
REACTOME_GPCR_LIGAND_BINDING	0.000
***Early Anti- and Anti-Inflammation***	
KEGG_OXIDATIVE_PHOSPHORYLATION	0.000
KEGG_RNA_DEGRADATION	0.001
KEGG_FATTY_ACID_METABOLISM	0.014
REACTOME_BRANCHED_CHAIN_AMINO_ACID_CATABOLISM	0.000
REACTOME_ELECTRON_TRANSPORT_CHAIN	0.000
REACTOME_INTEGRATION OF ENERGY METABOLISM	0.000
REACTOME_METABOLISM_OF_CARBOHYDRATES	0.000
REACTOME_PYRUVATE_METABOLISM_AND_TCA_CYCLE	0.000
REACTOME_METABOLISM_OF_PROTEIN	0.000
REACTOME_DIABETES_PATHWAYS	0.000
REACTOME_METABOLISM_OF_RNA	0.003
REACTOME_FORMATION_AND_MATURATION_OF_MRNA_TRANSCRIPTS	0.004
REACTOME_MRNA_SPLICING	0.009
REACTOME_METABOLISM_OF_MRNA	0.011
REACTOME_GENE_EXPRESSION	0.016
REACTOME_MICRORNA_BIOGENESIS	0.040
REACTOME_CELL_CYCLE_MITOTIC	0.000
REACTOME_G1_S_TRANSITION	0.000
REACTOME_G2_M_CHECKPOINTS	0.031

KEGG, Biocarta, and Reactome gene sets have been obtained from the C2: *curated gene sets collection* of the Molecular Signatures Database. Gene sets were defined as significantly enriched if FDR<0.05 when using Pearson as metric and 1,000 permutations of gene sets. The complete list of the Gene sets identified by GSEA is available with the authors.

### The M1 inflammatory signature develops into M2 during resolution

To assess the transition from M1 to M2 polarization, we merged 24 publicly available human microarray studies into a meta-dataset using A-MADMAN, and extracted gene expression data for 62 fresh monocyte samples, 46 M1 (treated with LPS/TNF-α or IFN-γ) and 20 M2 samples (M2c; treated with glucocorticoids, IL-10 or TGF-β). Gene expression signals of the meta-dataset were generated using the *Virtual-chip* approach that integrates raw expression data obtained from different Affymetrix arrays. The meta-dataset was analyzed with the SAM algorithm, to identify a list of genes differentially expressed in unstimulated monocytes, M1 and M2 macrophages.

**Figure 3:**
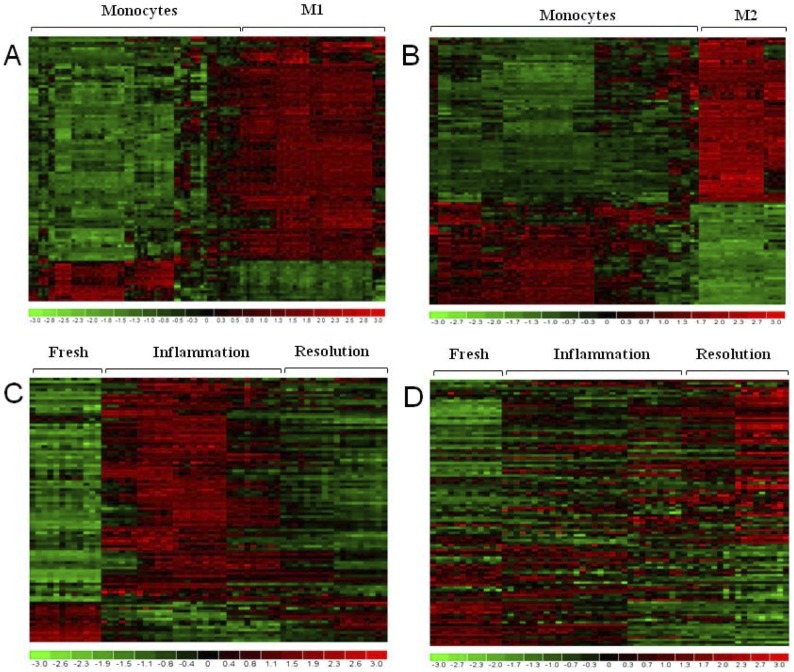
Differentially expressed genes in M1 and M2 macrophages *vs.* monocytes. Heat-maps representing the fold-expression levels of gene lists identified by SAM as statistically downregulated (green) or upregulated (red) in M1 and M2 samples compared to fresh unstimulated monocytes. The lists excluded genes that are modulated in both M1 and M2 *vs.* monocytes. (A) Fold-expression levels in monocytes and M1 macrophages of the meta-database for the 98 genes associated to monocyte-to-M1 differentiation. (B) Fold-expression levels in monocytes and M2 macrophages of the meta-database for the 107 genes associated to monocyte-to-M2 differentiation. (C) Fold-expression levels of the 98 monocyte-to-M1 genes assessed in the 60 samples of our *in vitro* model of inflammation. (D) Fold-expression levels of the 107 monocyte-to-M2 genes assessed in the 60 samples of our *in vitro* model of inflammation.

The statistical comparison returned that monocyte-to-M1 differentiation is associated with modulation of 98 genes, of which 85% are highly expressed in M1 and 15% in monocytes (Figure 3A, Table S4), while monocyte-to-M2 differentiation resulted in the modulation of 107 genes, 62% highly expressed in M2 and 38% in monocytes (Figure 3B, Table S5). Transcripts that are upregulated in M1 cells *vs.* monocytes included cytokines and chemokines, while those upregulated in M2 cells included enzymes and extracellular mediators. The two signatures of M1 and M2 polarization were used to cluster samples of our *in vitro* model of inflammation. As shown in the Figure 3C, fresh monocytes showed a gene expression profile fully overlapping with that of unstimulated monocytes in the meta-database, then they presented a M1-like expression profile during the inflammatory phases, to return to a monocyte-like profile in the resolution phase. When considering the gene set that discriminates monocytes from M2 cells, fresh monocytes showed the same profile as the untreated monocytes of the meta-database, and this profile gradually changed during the progression of inflammation, to become similar to that of M2 macrophages at the end of resolution phase (Figure 3D).

When comparing the list of genes differentially expressed during the inflammation process (Figure 2) with the list of genes differentially expressed in monocytes *vs.* M1 (Figure 3A), a large number of genes expressed in M1 cells (34%) belong to the *Inflammation* group. Conversely, 21% of genes expressed in M2 cells belong to the *Positive Differentiation* group and are expressed only during the resolution phase. In the monocytes *vs.* M1 comparison, a large part of genes expressed in fresh monocytes belongs to the *Anti-Inflammation* group (26%), while in the monocytes *vs.* M2 comparison 51% of genes expressed in monocytes are in the *Negative Differentiation* group. Among genes common to both M1 and M2 polarization, several belong to the *Inflammation Driven Differentiation* group (14% and 20%, respectively). Table 2 shows some representative genes identified in these comparisons.

**Table 2:** Correlation between M1/M2 polarization and functional groups.

Gene Symbol	Functional Groups
**Genes upregulated in M1 polarization**	
*IL12B, PTX3, CCL4, IL1RN, TNF, IL6, CCL20, IL1A, ICAM1, NFKB1, TRAF1, SERPINB9, IL1F9, MAFF*	*Inflammation*
*CXCL1, DRAM, TNIP3, CCL2, SLAMF7, CCR7, TNFAIP6*	*Inflammation Driven Differentiation*
**Genes downregulated in M1 polarization**	
*P2RY5, FGL2, CD1D*	*Anti-Inflammation*
**Genes upregulated in M2 polarization**	
*TREM2, A2M, NUPR1, C1QA, MS4A4A, APOE, APOC1, ADORA3*	*Positive Differentiation*
*ADAMDEC1, CD59, TFPI, CCL3*	*Inflammation Driven Differentiation*
**Genes downregulated in M2 polarization**	
*FCER1A, LGALS2, PF4, CD69, CD93, NR4A2, VCAN, CD62L, ICAM3, NLRP3, ERG1*	*Negative Differentiation*

Association of gees that are up- or downregulated in M1 and M2 cells polarisation (Figure 3A and B) with the functional groups defined from the analysis of the *in vitro* model of inflammation (Figure 2).

### qRT-PCR and ELISA validation

**Figure 4:**
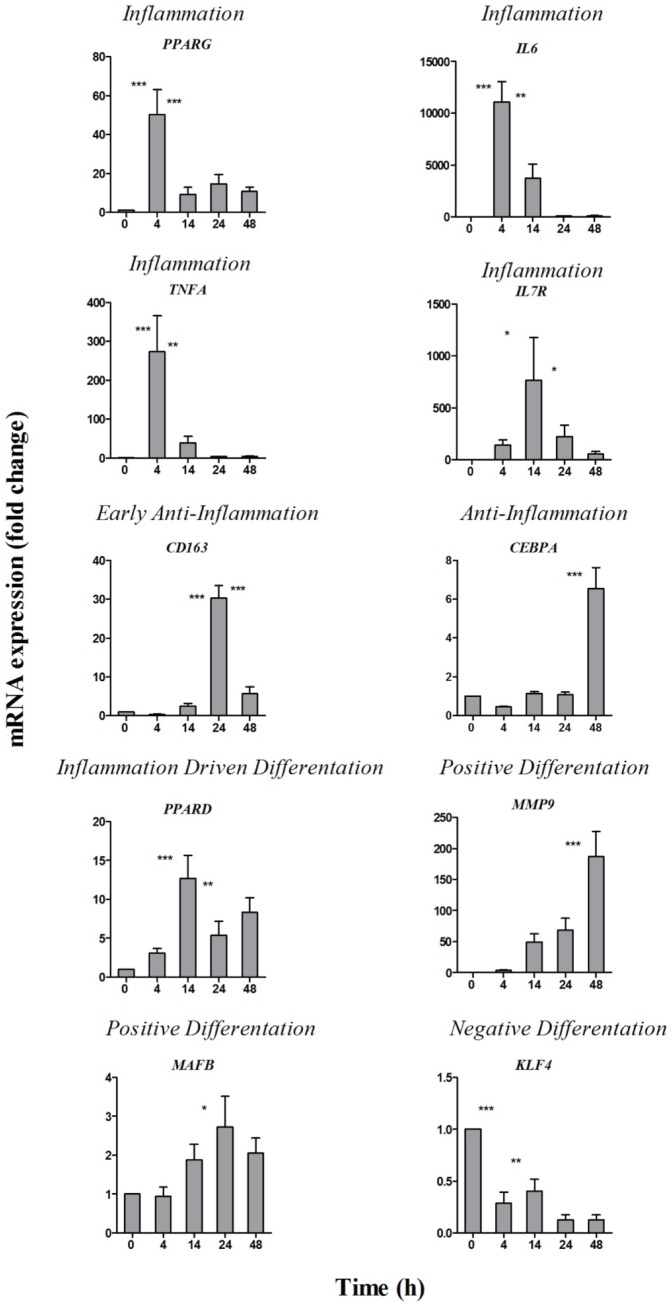
Gene expression validation by qPCR. Fold-expression levels determined by qPCR for the 10 genes selected in the *Inflammation* (*PPARG*, *IL6*, *TNFA*, *IL7R*), *Early Anti-Inflammation* (*CD163*), *Anti-Inflammation* (*CEBPA*), *Inflammation Driven Differentiation* (*PPARD*), *Positive Differentiation* (*MMP9* and *MAFB*), and *Negative Differentiation* (*KLF4*) groups. The mean expression values ± SEM from six different donors are reported. Statistical significance was calculated with ANOVA followed by Fisher's test for significant differences between two consecutive experimental time points. * *P*<.05; ** *P*<.001; *** *P*<.0001.

A subset of ten genes was assessed by qRT-PCR, employing the same RNA samples used to perform microarray experiments, five transcription factors chosen as markers of monocyte differentiation, and five inflammation-related factors as markers of monocyte activation, selected within each functional group of Figure 2. The qPCR results confirmed the expression patterns observed by microarray analysis (Figure 4). Genes belonging to the *Inflammation* group (*PPARG*, *IL6*, *TNFA*) were upregulated during the early phase, while *IL7R* was over-expressed during the late phase of inflammation. *CD163* (*Early Anti-Inflammation*) was highly upregulated at the beginning of resolution, possibly induced by IL-10, while the transcription factor *CEBPA* (*Anti-Inflammation*) was overexpressed during late resolution, possibly induced by TGF-β. Expression of *PPARD* (*Inflammation Driven Differentiation*) increased during late inflammation and remained elevated, while *MAFB* and *MMP9* genes (*Positive Differentiation*) were upregulated during resolution. Finally, expression of *KLF4* (*Negative Differentiation*) was high in fresh monocytes and decreased thereafter.

**Figure 5:**
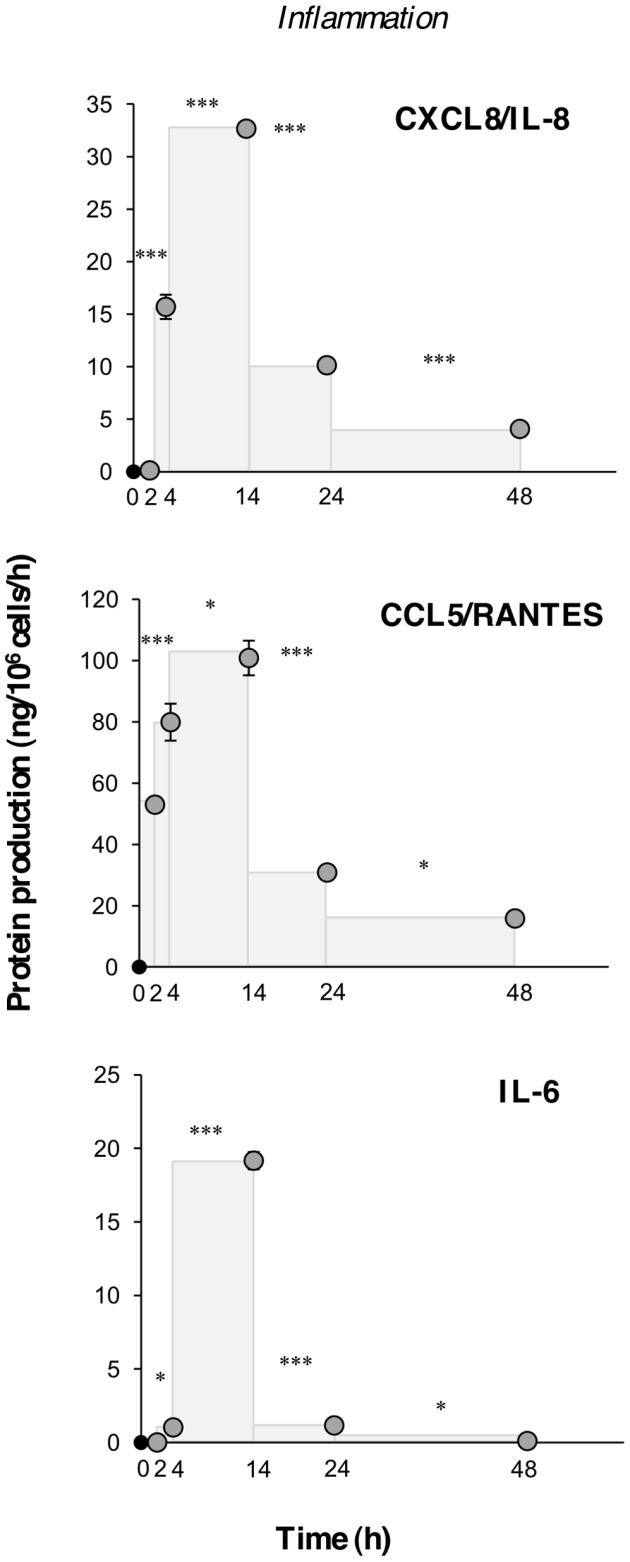
Rate of cytokine and chemokine production in the course of *in vitro* inflammation. Production of inflammatory chemokines CXCL8 (IL-8) and CCL5 (RANTES), and of the cytokine IL-6 during the *in vitro* inflammatory reaction. Production of soluble proteins released in the supernatant is reported in terms of rate of production, *i.e.*, the amount of protein produced per one million cells per hour. The mean values ± SEM of three different donors are reported. Statistical significance was calculated with ANOVA followed by Fisher's test for significant differences between two consecutive experimental time-points. * *P*<.05; ** *P*<.001; *** *P*<.0001.

Production and secretion of the inflammatory cytokine IL-6 and of the M1 polarization-associated chemokines CXCL8 (IL-8) and CCL5 (RANTES) were evaluated in terms of rate of production and resulted abundantly produced during the inflammatory phase, to be turned off during resolution (Figure 5). Production of CCL5 was already significant after stimulation with CCL2 only, in agreement with previous findings that CCL2 can induce chemokine production in monocytes [31].

## Discussion

In this study, we set up an *in vitro* model of inflammation based on human primary monocytes with the aim of describing the kinetics of the inflammatory reaction, from initiation and development until eventual resolution. The use of normal human cells allowed us to study the mechanisms of inflammation in a system that readily translates to human responsiveness *in vivo*, as opposed to mouse models [32], [33] or transformed cell systems [34], [35]. Transcriptomic profiling was performed on monocytes from 12 healthy donors, selected over a large age range (19–56 years), including both females and males, and encompassing individuals of three different ethnic groups. This heterogeneity is expected to capture, at least partially, the human variability. Despite such heterogeneity, the gene expression profiles of the donors appeared to be very reproducible, both in fresh monocytes and in response to all the stimulation conditions at the different time points, suggesting that the monocyte response is highly reliable and robust. It should be said that additional experiments (where expression of a limited number of gene was performed by real-time PCR) have brought the number of donors to over 60, and we never observed significant discrepancies in the monocyte type and kinetics of response (Italiani *et al.*, unpublished). Also, the use of viral stimuli instead of LPS, besides triggering a virus-specific signature, did essentially bring about a response that largely overlapped that described in this study (Boraschi *et al.*, unpublished).

The *in vitro* model of inflammation presented here aims at representing, in a simplified fashion, the course of an inflammatory reaction in a tissue, limited to the role of newly recruited inflammatory blood monocytes. In fact, the simplified model does not include resident tissue macrophages, highly differentiated cells with a clear M2 bias, that are the cells initiating inflammation and responsible for the recruitment of blood monocytes, nor other cell types. Also, the role of extracellular matrix components and of the tissue architecture is not considered in this model. Although there is some evidence that EMC do not affect macrophages polarization [36], it is likely that the structure of the tissue environment has a role in determining the response outcome. More complex models, to reproduce the inflammatory response of both infiltrating monocytes and resident macrophages in specific tissues, are underway in our lab. Taking in consideration the limitations of the present model, its advantages are its simplicity and robustness (with responses that are the same in different donors and in different stimulation conditions), its accurate kinetic description of monocyte inflammatory reaction and the passage from monocytes to M1 and then to M2, and its higher validity in describing human inflammation, as opposed to the use of transformed cell lines or animal cells.

Many genes were differentially expressed throughout the inflammatory reaction and the concomitant monocyte-to-macrophage differentiation. Homogeneity of gene expression profiles among different donors underlines the robustness of the model. Supervised hierarchical clustering allowed defining five major functional groups of modulated genes. The *Inflammation* cluster, corresponding to monocyte-to-M1 differentiation, includes genes encoding classical inflammatory effectors, such as inflammatory cytokines (*IL1B*, *IL6*, *TNFA*, *IL12B*), chemokines (*CXCL8*, *CCL5*, *CCL20*), soluble innate mediators (*PTX3*, *EDN1*, *APOL2*), and enzymes (*PTGS2*, *PLA1A*). *Early Anti-Inflammation* and *Anti-Inflammation* include genes downregulated in M1 polarised cells, *i.e.*, genes encoding transcriptional factor such as CCAAT/enhancer binding protein alpha (*CEBPA*), innate receptors (*TLR5*, *TLR7*, *TLR8*), purinergic receptors (*P2RX7*), FcR (*FCER1A*, *FCRLB*), and metallothionein genes (*MT1G*, *MT4*, *MT1E*, *MT1M*, *MT1F*, *MT1X*), involved in modulation of inflammation, control of the oxidative stress, cell proliferation [37], and strongly upregulated in endotoxin tolerance [38]. The decreased expression of inflammatory receptor genes may be related to loss of responsiveness following activation (similar to tolerance), which is restored at the end of inflammatory process when inflammatory monocytes have become tissue-regulating macrophages ready to respond to a new danger signal.

*Inflammation Driven Differentiation* encompasses genes whose expression rapidly increased at the onset of inflammation and remained upregulated throughout. These genes may be needed both for the inflammatory response and for monocyte differentiation into tissue-repairing macrophages. Indeed, this cluster includes inflammatory genes and M1 polarization markers (*IL7R*, *CCR7*, *CCL19*, *CXCL11*), and several genes highly expressed in M2c polarization (*IL10*, *CCL24*, *CCL22*). *Positive* and *Negative Differentiation* include genes important for monocyte-to-macrophage differentiation, such as transcription factors (*MAFB*, *KLF4*, *PPARG*), c-type lectins (*CLEC3B*, *CLEC7A*, *CLEC10A*, *CLEC11A*), adhesion (*SELL*, *ICAM3*, *AMICA1*) and signalling molecules (MAP kinases), and extracellular mediators (*C1Q*, *APOE*). These genes may define the differentiation of monocytes to macrophages independently of the concurring inflammatory reaction. Indeed, monocytes used in these experiments are a heterogeneous population as present in the blood and could therefore include both “inflammatory” monocytes differentiating into effector cells in the tissue, and “homeostatic” monocytes replenishing the pool of tissue macrophages in physiological conditions [6], [39].

The majority of pathways identified in the *Inflammation* cluster are involved in innate immune activation and type I inflammation (NFκB, MAPK and JAK-STAT signalling, NLR and TLR signalling, cytokine/chemokine receptor interaction, IL-1R pathway), while the *Early Anti-Inflammation and Anti-Inflammation* clusters are enriched in pathways associated to lipid, protein, and carbohydrate metabolism, regulation of gene expression (RNA splicing and miRNA biogenesis), and cell cycle. The same pathways were found in the *Positive Differentiation* cluster. The modulation of genes involved in cellular metabolic activities is a prominent feature of M2 macrophage polarization/differentiation [40]–[42], and during the resolution and repair phases, when major rearrangements of cellular functions are required for shifting from inflammation to anti-inflammation and tissue repair. The enrichment in pathways associated with cell cycle agrees with previous observations [43], and underlines the importance of proliferation in M2-polarised macrophages [44], although its *in vivo* relevance is still debated. By comparing the genes differentially expressed between monocytes *vs.* M1 and *vs.* M2, it is evident that monocytes in our model show an M1 signature in the inflammatory phase and an M2 profile during resolution. Most genes expressed in M1 belong to the *Inflammation* cluster, while those in M2 belong to *Positive Differentiation*, and several genes related to both M1 and M2 polarization belong to *Inflammation Driven Differentiation*. That this latter cluster is related to both activation programs suggests that inflammation is a process strictly connected to macrophage differentiation. Thus, that the genes involved in inflammatory activation belong to the same biological pathways involved in cellular processes of monocyte-to-macrophage differentiation establishes a transcriptional connection between monocyte activation and differentiation, inflammation and metabolism. Therefore, resolution of inflammation is strictly connected to macrophage differentiation in the tissue.

These observations suggest that monocytes entering an inflammatory environment first polarise into M1, and then switch to M2 upon microenvironmental changes. The fact that the same monocyte population goes through all the phases of the inflammatory process by adapting its phenotype and function to the evolution of microenvironmental conditions was already suggested by studies in mouse models [11]–[13], but never previously shown for human cells.

The shift from M1 to M2 was confirmed by assessing quantitative gene expression and protein production for a series of cytokines, markers and transcriptional factors involved in both monocyte differentation and macrophage polarization. Our data confirmed that expression of *MAFB*, a myeloid differentiation marker, correlates with expression of its target genes *CD163* and *MMP9* [45], which increase during the resolution phase. Expression of *PPARG* and *PPARD* increased during the inflammation phases (*PPARD* maintaining high expression also during resolution), confirming their role in inflammation [46], and in control of monocyte-to-macrophage differentiation [47], respectively. *KLF4* and *CEBPA*, critical regulators of monocyte differentiation, showed an opposite expression profile, the former being significantly downregulated throughout, while the latter was strongly upregulated during late resolution. The observed *PPARG* and *KLF4* expression profiles do not agree with the reported observation that these factors are linked to M2 polarization [48]. However, previous studies addressed M2a polarization (type II inflammation), at variance with our model exclusively focused on M2c polarization (deactivation), which is functionally very different. Thus, while transcriptional factors may variously contribute to macrophage polarization, downregulation of *PPARG* and *KLF4* in parallel to upregulation of *MAFB* seem to be critical for monocyte to M2c differentiation.

Overall, this study shows that an *in vitro* system based on primary human cells can allow us to describe the kinetic development of cell reactivity and its modulation during the entire course of the inflammatory response in a robust and reliable fashion. The use of such human primary cell-based models are bound to provide information readily transferrable to human reactivity *in vivo*, and to identify regulatory pathways associated with physiological response or with persistent and pathological inflammation.

## Supporting Information

Figure S1 **Data visualization by cluster analysis.** Nine separated clusters are shown. Solid red lines have been drawn joining the average value of gene expression at each time point for each donor (dots). In the text the clusters are reported as follows: 1 and 2 as *Inflammation* (218 and 174 genes, respectively), 3 as *Early Anti-Inflammation* (850 genes), 4 and 5 as *Anti-Inflammation* (445 and 576 genes respectively), 6 as *Inflammation Driven Differentiation* (457 genes), 7 as *Positive Differentiation* (214 genes), 8 and 9 as *Negative Differentiation* (680 and 381 genes, respectively).
(TIFF)

Table S1 **Complete list of the datasets used in this study and their sources.** Genome-wide expression levels and meta-information of 303 samples were organized in a proprietary database using A-MADMAN.
(DOCX)

Table S2 **Complete list of 128 samples labeled as untreated monocytes and as M1 and M2 activated monocytes and their sources.**
(DOCX)

Table S3 **Complete list of genes differentially expressed during the course of the in vitro inflammatory reaction.** NA: not attributed
(XLS)

Table S4 **Complete list of the genes differentially expressed between untreated monocytes and M1 macrophages, extracted from database.**
(DOCX)

Table S5 **Complete list of the genes differentially expressed between untreated monocytes and M2 macrophages, extracted from database.**
(DOCX)
