# Supplementary figures and images for: Transcriptomic Profiling of the Development of the Inflammatory Response in Human Monocytes In Vitro
Source: PLoS One. 2014 Feb 3;9(2):e87680. doi: 10.1371/journal.pone.0087680 (PMC3912012; doi:10.1371/journal.pone.0087680)

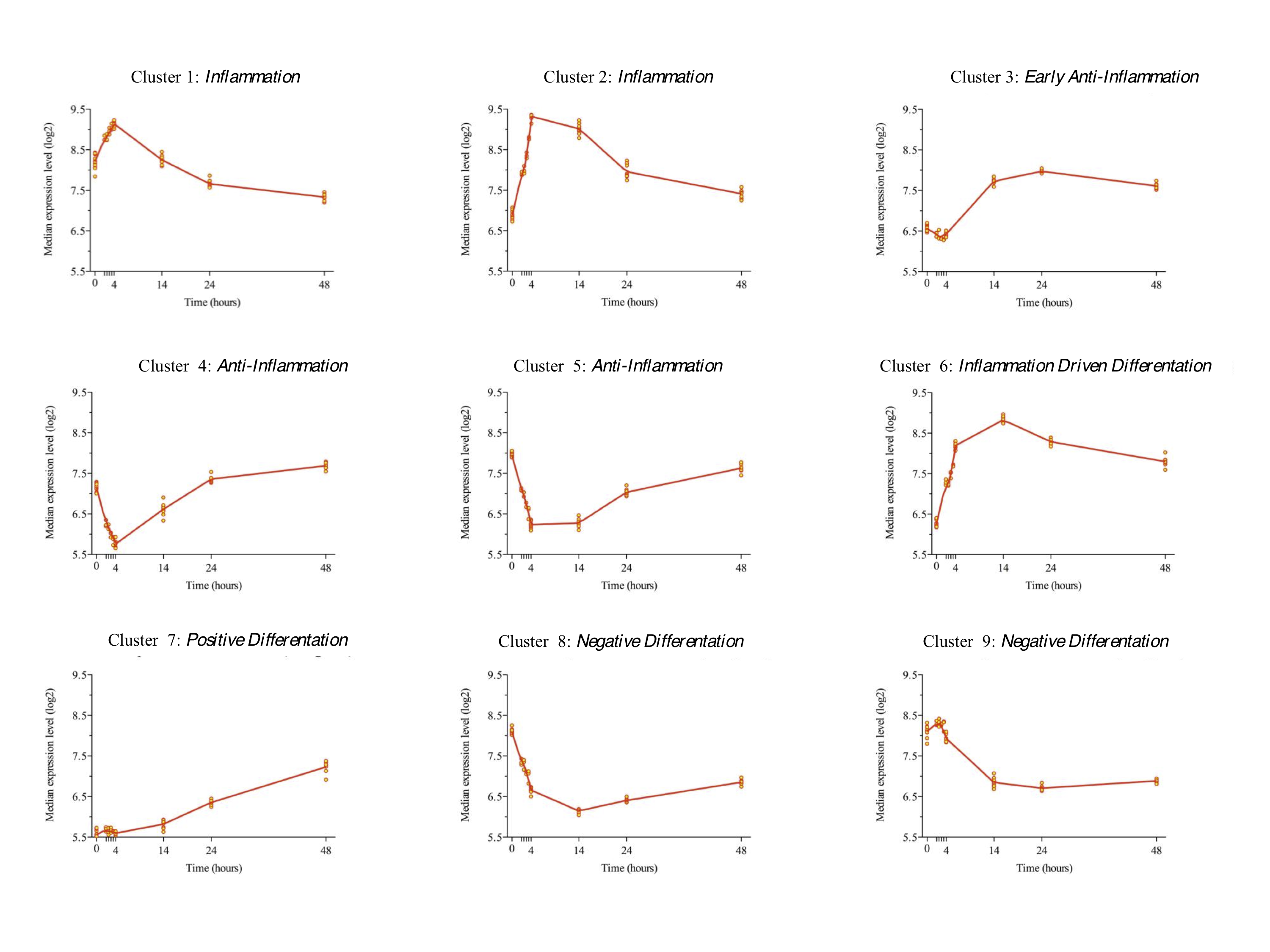

Supplement: Figure S1 — Data visualization by cluster analysis. Nine separated clusters are shown. Solid red lines have been drawn joining the average value of gene expression at each time point for each donor (dots). In the text the clusters are reported as follows: 1 and 2 as Inflammation (218 and 174 genes, respectively), 3 as Early Anti-Inflammation (850 genes), 4 and 5 as Anti-Inflammation (445 and 576 genes respectively), 6 as Inflammation Driven Differentiation (457 genes), 7 as Positive Differentiation (214 genes), 8 and 9 as Negative Differentiation (680 and 381 genes, respectively). (TIFF) [file pone.0087680.s001.tiff]
