# Supplementary material for: Transcriptomic Profiling of the Development of the Inflammatory Response in Human Monocytes In Vitro
Source: PLoS One. 2014 Feb 3;9(2):e87680. doi: 10.1371/journal.pone.0087680 (PMC3912012; doi:10.1371/journal.pone.0087680)
Supplement: Table S1 — Complete list of the datasets used in this study and their sources. Genome-wide expression levels and meta-information of 303 samples were organized in a proprietary database using A-MADMAN. (DOCX) [file pone.0087680.s002.docx]

**Table S1**

| **GEO series** | **Platform** | **Total samples in series** | **Samples used in this study** | **Reference**^a^ |
| --- | --- | --- | --- | --- |
| GSE4984 | HG-U133 Plus2.0 | 12 | 6 | Fulcher et al., 2006 |
| GSE5099 | HG-U133A | 30 | 14 | Martinez et al., 2006 |
| GSE5547 | HG-U133 Plus2.0 | 24 | 6 | Humphrey et al., 2007 |
| GSE6965 | HG-U133 Plus2.0 | 4 | 4 | Mezger et al., 2008 |
| GSE7509 | HG-U133 Plus2.0 | 26 | 26 | Dhodapkar et al., 2007 |
| GSE7568 | HG-U133 Plus2.0 | 25 | 25 | Gratchev et al., 2008 |
| GSE7807 | HG-U133 Plus2.0 | 8 | 4 | Woszczek et al., 2008 |
| GSE8286 | HG-U133A | 9 | 9 | Liu et al., 2008 |
| GSE8515 | HG-U133A | 15 | 15 | Jura et al., 2008 |
| GSE8608 | HG-U133 Plus2.0 | 6 | 1 | Hofer et al., 2008 |
| GSE8658 | HG-U133 Plus2.0 | 63 | 30 | Szatmari et al., 2007 |
| GSE9080 | HG-U133Av2 | 6 | 3 | --- |
| GSE9874 | HG-U133A | 60 | 11 | Hägg et al., 2008 |
| GSE9946 | HG-U133A | 12 | 12 | Popov et al., 2008 |
| GSE9988 | HG-U133 Plus2.0 | 62 | 58 | Dower et al., 2008 |
| GSE10856 | HG-U133 Plus2.0 | 4 | 4 | Chang et al., 2008 |
| GSE11393 | HG-U133Av2 | 9 | 3 | Llaverias et al., 2008 |
| GSE11430 | HG-U133 Plus2.0 | 10 | 10 | Maouche et al., 2008 |
| GSE11864 | HG-U133 Plus2.0 | 10 | 10 | Hu et al., 2008 |
| GSE12108 | HG-U133 Plus2.0 | 14 | 13 | Butchar et al., 2008 |
| GSE12773 | HG-U133 Plus2.0 | 10 | 5 | Rate et al., 2009 |
| GSE12837 | HG-U133A | 24 | 3 | Coppe et al., 2009 |
| GSE13762 | HG-U133 Plus2.0 | 15 | 15 | Széles et al., 2009 |
| GSE14419 | HG-U133Av2 | 16 | 16 | --- |

**^a^References**

Butchar JP, Cremer TJ, Clay CD et al. Microarray analysis of human monocytes infected with Francisella tularensis identifies new targets of host response subversion. *PLoS One*. 2008;3(8):e2924.

Chang YC, Chen TC, Lee CT et al. Epigenetic control of MHC class II expression in tumor-associated macrophages by decoy receptor 3. *Blood*. 2008;111(10):5054-5063.

Coppe A, Ferrari F, Bisognin A et al. Motif discovery in promoters of genes co-localized and co-expressed during myeloid cells differentiation. *Nucleic Acids Res*. 2009;37(2):533-549.

Dhodapkar KM, Banerjee D, Connolly J et al.. Selective blockade of the inhibitory Fcgamma receptor (FcgammaRIIB) in human dendritic cells and monocytes induces a type I interferon response program. *J Exp Med*. 2007;204(6):1359-1369.

Dower K, Ellis DK, Saraf K et al. Innate immune responses to TREM-1 activation: overlap, divergence, and positive and negative cross-talk with bacterial lipopolysaccharide. *J Immunol*. 2008;180(5):3520-3534.

Fulcher JA, Hashimi ST, Levroney EL et al. Galectin-1-matured human monocyte-derived dendritic cells have enhanced migration through extracellular matrix. *J Immunol*. 2006;177(1):216-226.

Gratchev A, Kzhyshkowska J, Kannookadan S et al. Activation of a TGF-beta-specific multistep gene expression program in mature macrophages requires glucocorticoid-mediated surface expression of TGF-beta receptor II. *J Immunol*. 2008;180(10):6553-6565.

Hägg DA, Jernås M, Wiklund O et al. Expression profiling of macrophages from subjects with atherosclerosis to identify novel susceptibility genes. *Int J Mol Med*. 2008;21(6):697-704.

Hofer TP, Frankenberger M, Mages J et al. Tissue-specific induction of ADAMTS2 in monocytes and macrophages by glucocorticoids. *J Mol Med*. (Berl) 2008;86(3):323-332.

Hu X, Chung AY, Wu I et al. Integrated regulation of Toll-like receptor responses by Notch and interferon-gamma pathways. *Immunity*. 2008;29(5):691-703.

Humphreys TL, Li L, Li X et al. Dysregulated immune profiles for skin and dendritic cells are associated with increased host susceptibility to Haemophilus ducreyi infection in human volunteers. *Infect Immun*. 2007;75(12):5686-5697.

Jura J, Wegrzyn P, Korostyński M et al. Identification of interleukin-1 and interleukin-6-responsive genes in human monocyte-derived macrophages using microarrays. *Biochim Biophys Acta*. 2008;1779(6-7):383-389.

Liu H, Shi B, Huang CC et al. Transcriptional diversity during monocyte to macrophage differentiation. *Immunol Lett*. 2008;117(1):70-80.

Llaverias G, Pou J, Ros E et al. Monocyte gene-expression profile in men with familial combined hyperlipidemia and its modification by atorvastatin treatment. *Pharmacogenomics*. 2008;9(8):1035-1054.

Maouche S, Poirier O, Godefroy T et al. Performance comparison of two microarray platforms to assess differential gene expression in human monocyte and macrophage cells. *BMC Genomics*. 2008;9:302.

Martinez FO, Gordon S, Locati M et al. Transcriptional profiling of the human monocyte-to-macrophage differentiation and polarization: new molecules and patterns of gene expression. *J Immunol*. 2006;177(10):7303-7311.

Mezger M, Wozniok I, Blockhaus C et al. Impact of mycophenolic acid on the functionality of human polymorphonuclear neutrophils and dendritic cells during interaction with Aspergillus fumigatus. *Antimicrob Agents Chemother*. 2008;52(7):2644-2646.

Popov A, Driesen J, Abdullah Z et al. Infection of myeloid dendritic cells with Listeria monocytogenes leads to the suppression of T cell function by multiple inhibitory mechanisms. *J Immunol*. 2008;181(7):4976-4988.

Rate A, Upham JW, Bosco A, McKenna KL, Holt PG. Airway epithelial cells regulate the functional phenotype of locally differentiating dendritic cells: implications for the pathogenesis of infectious and allergic airway disease. *J Immunol*. 2009;182(1):72-83.

Szatmari I, Töröcsik D, Agostini M et al. PPARgamma regulates the function of human dendritic cells primarily by altering lipid metabolism. *Blood*. 2007;110(9):3271-3280.

Széles L, Keresztes G, Töröcsik D et al. 1,25-dihydroxyvitamin D3 is an autonomous regulator of the transcriptional changes leading to a tolerogenic dendritic cell phenotype. *J Immunol*. 2009;182(4):2074-2083

Woszczek G, Chen LY, Nagineni S et al. Leukotriene D(4) induces gene expression in human monocytes through cysteinyl leukotriene type I receptor. *J Allergy Clin Immunol*. 2008;121(1):215-221.e1
