# Supplementary material for: Transcriptomic Profiling of the Development of the Inflammatory Response in Human Monocytes In Vitro
Source: PLoS One. 2014 Feb 3;9(2):e87680. doi: 10.1371/journal.pone.0087680 (PMC3912012; doi:10.1371/journal.pone.0087680)
Supplement: Table S2 — Complete list of 128 samples labeled as untreated monocytes and as M1 and M2 activated monocytes and their sources. (DOCX) [file pone.0087680.s003.docx]

**Table S2**

| **GEO series** | **Platform** | **GEO samples** |
| --- | --- | --- |
| *Untreated monocytes* | | |
| GSE5099 | HG-U133A | GSM115051; GSM115046; GSM115047; GSM115048; GSM115049; GSM115050 |
| GSE7807 | HG-U133 Plus2.0 | GSM189447; GSM189448; GSM189449; GSM189450 |
| GSE8286 | HG-U133A | GSM205587; GSM205588; GSM205590; GSM205591; GSM205592; GSM205594 |
| GSE8658 | HG-U133 Plus2.0 | GSM214749; GSM214734; GSM214737; GSM214738; GSM214739; GSM214740; GSM214741; GSM214742; GSM214743; GSM214744; GSM214745; GSM214746 |
| GSE9080 | HG-U133Av2 | GSM230145; GSM230149; GSM230147 |
| GSE9988 | HG-U133 Plus2.0 | GSM252476; GSM252478; GSM252479; GSM252480; GSM252481; GSM252484; GSM252485 |
| GSE11393 | HG-U133Av2 | GSM287664; GSM287665; GSM287666 |
| GSE11430 | HG-U133 Plus2.0 | GSM257664; GSM257666; GSM257668; GSM257670; GSM257672 |
| GSE11864 | HG-U133 Plus2.0 | GSM299556; GSM299557; GSM299561; GSM299562 |
| GSE12108 | HG-U133 Plus2.0 | GSM305434; GSM305436; GSM305438; GSM305440; GSM305430; GSM305432 |
| GSE12837 | HG-U133A | GSM15431; GSM321582; GSM15430 |
| GSE13762 | HG-U133 Plus2.0 | GSM346564; GSM346577; GSM346553 |
| *M1 activation* | | |
| GSE5099 | HG-U133A | GSM115055; GSM115057 |
| GSE9988 | HG-U133 Plus2.0 | GSM252423; GSM252424; GSM252425; GSM252427; GSM252428; GSM252429; GSM252431; GSM252432; GSM252433; GSM252434; GSM252435; GSM252436; GSM252437; GSM252438; GSM252439; GSM252440; GSM252441; GSM252442; GSM252443; GSM252444; GSM252445; GSM252447; GSM252448; GSM252449; GSM252450; GSM252451; GSM252453; GSM252454; GSM252455; GSM252456; GSM252457; GSM252458; GSM252459; GSM252460; GSM252461; GSM252462; GSM252463; GSM252464; GSM252430; GSM252426 |
| GSE14419 | HG-U133Av2 | GSM360141; GSM360145; GSM360184; GSM360188 |
| *M2 activation* | | |
| GSE7568 | HG-U133 Plus2.0 | GSM183464; GSM183465; GSM183466; GSM183467; GSM183482; GSM183483; GSM183484; GSM183485; GSM183486; GSM183487; GSM183217; GSM183305; GSM183306; GSM183315; GSM183316; GSM183392; GSM183393; GSM183394; GSM183462; GSM183463 |
