# Supplementary material for: Transcriptomic Profiling of the Development of the Inflammatory Response in Human Monocytes In Vitro
Source: PLoS One. 2014 Feb 3;9(2):e87680. doi: 10.1371/journal.pone.0087680 (PMC3912012; doi:10.1371/journal.pone.0087680)
Supplement: Table S4 — Complete list of the genes differentially expressed between untreated monocytes and M1 macrophages, extracted from database. (DOCX) [file pone.0087680.s005.docx]

**Table S4**

| Gene Id | **Symbol** | **Description** |
| --- | --- | --- |
| GC08P019841_at | LPL | lipoprotein lipase |
| GC04P089115_at | SPP1 | secreted phosphoprotein 1 |
| GC08P081561_at | ZBTB10 | zinc finger and BTB domain containing 10 |
| GC05M158674_at | IL12B | interleukin 12B (natural killer cell stimulatory factor 2, cytotoxic lymphocyte maturation factor 2, p40) |
| GC08P086563_at | CA2 | carbonic anhydrase II |
| GC08M105570_at | LRP12 | low density lipoprotein-related protein 12 |
| GC19M006615_at | TNFSF14 | tumor necrosis factor (ligand) superfamily, member 14 |
| GC05M147184_at | SPINK1 | serine peptidase inhibitor, Kazal type 1 |
| GC09M116591_at | TNFSF15 | tumor necrosis factor (ligand) superfamily, member 15 |
| GC11M102146_at | MMP10 | matrix metallopeptidase 10 (stromelysin 2) |
| GC04P074974_at | CXCL1 | chemokine (C-X-C motif) ligand 1 (melanoma growth stimulating activity, alpha) |
| GC12M010202_at | OLR1 | oxidized low density lipoprotein (lectin-like) receptor 1 |
| GC05P149320_at | SLC26A2 | solute carrier family 26 (sulfate transporter), member 2 |
| GC12P027288_at | STK38L | serine/threonine kinase 38 like |
| GC12M088484_at | ATP2B1 | ATPase, Ca++ transporting, plasma membrane 1 |
| GC19P054067_at | PPP1R15A | protein phosphatase 1, regulatory (inhibitor) subunit 15A |
| GC03P158637_at | PTX3 | pentraxin-related gene, rapidly induced by IL-1 beta |
| GC20M043387_at | SDC4 | syndecan 4 |
| GC11M002906_at | PHLDA2 | pleckstrin homology-like domain, family A, member 2 |
| GC01P239781_at | KMO | kynurenine 3-monooxygenase (kynurenine 3-hydroxylase) |
| GC02P187163_at | ITGAV | integrin, alpha V (vitronectin receptor, alpha polypeptide, antigen CD51) |
| GC01P078182_at | DNAJB4 | DnaJ (Hsp40) homolog, subfamily B, member 4 |
| GC06P012120_at | HIVEP1 | human immunodeficiency virus type I enhancer binding protein 1 |
| GC02P191222_at | NAB1 | NGFI-A binding protein 1 (EGR1 binding protein 1) |
| GC03M195606_at | ATP13A3 | ATPase type 13A3 |
| GC07P065308_at | TPST1 | tyrosylprotein sulfotransferase 1 |
| GC01M094706_at | F3 | coagulation factor III (thromboplastin, tissue factor) |
| GC01M177339_at | ABL2 | v-abl Abelson murine leukemia viral oncogene homolog 2 (arg, Abelson-related gene) |
| GC07M041695_at | INHBA | inhibin, beta A |
| GC17P031421_at | CCL4 | chemokine (C-C motif) ligand 4 |
| GC02P113591_at | IL1RN | interleukin 1 receptor antagonist |
| GC07P100558_at | SERPINE1 | serpin peptidase inhibitor, clade E (nexin, plasminogen activator inhibitor type 1), member 1 |
| GC17P029621_at | CCL7 | chemokine (C-C motif) ligand 7 |
| GC08M095330_at | GEM | GTP binding protein overexpressed in skeletal muscle |
| GC12M074707_at | PHLDA1 | pleckstrin homology-like domain, family A, member 1 |
| GC11M008960_at | NRIP3 | nuclear receptor interacting protein 3 |
| GC16M086421_at | SLC7A5 | solute carrier family 7 (cationic amino acid transporter, y+ system), member 5 |
| GC09P101623_at | NR4A3 | nuclear receptor subfamily 4, group A, member 3 |
| GC0XP149282_at | MAMLD1 | mastermind-like domain containing 1 |
| GC06P031652_at | TNF | tumor necrosis factor (TNF superfamily, member 2) |
| GC07P022732_at | IL6 | interleukin 6 (interferon, beta 2) |
| GC16P082737_at | LRRC50 | leucine rich repeat containing 50 |
| GC09P000461_at | KANK1 | KN motif and ankyrin repeat domains 1 |
| GC08M080838_at | HEY1 | hairy/enhancer-of-split related with YRPW motif 1 |
| GC02P228386_at | CCL20 | chemokine (C-C motif) ligand 20 |
| GC02M113247_at | IL1A | interleukin 1, alpha |
| GC11M064376_at | EHD1 | EH-domain containing 1 |
| GC22P022997_at | ADORA2A | adenosine A2a receptor |
| GC19P010247_at | ICAM1 | intercellular adhesion molecule 1 |
| GC01P037712_at | ZC3H12A | zinc finger CCCH-type containing 12A |
| GC06M143114_at | HIVEP2 | human immunodeficiency virus type I enhancer binding protein 2 |
| GC04P103641_at | NFKB1 | nuclear factor of kappa light polypeptide gene enhancer in B-cells 1 |
| GC09M122704_at | TRAF1 | TNF receptor-associated factor 1 |
| GC02P151922_at | TNFAIP6 | tumor necrosis factor, alpha-induced protein 6 |
| GC08M072916_at | MSC | musculin (activated B-cell factor-1) |
| GC17P071890_at | SPHK1 | sphingosine kinase 1 |
| GC20M055657_at | PMEPA1 | prostate transmembrane protein, androgen induced 1 |
| GC01M207854_at | LAMB3 | laminin, beta 3 |
| GC06M002832_at | SERPINB9 | serpin peptidase inhibitor, clade B (ovalbumin), member 9 |
| GC16M065513_at | RRAD | Ras-related associated with diabetes |
| GC01P190871_at | RGS13 | regulator of G-protein signaling 13 |
| GC01P160797_at | UAP1 | UDP-N-acteylglucosamine pyrophosphorylase 1 |
| GC20M010566_at | JAG1 | jagged 1 (Alagille syndrome) |
| GC04M100046_at | EIF4E | eukaryotic translation initiation factor 4E |
| GC17P065677_at | KCNJ2 | potassium inwardly-rectifying channel, subfamily J, member 2 |
| GC12P100795_at | DRAM | damage-regulated autophagy modulator |
| GC14M050170_at | SAV1 | salvador homolog 1 (Drosophila) |
| GC04M122332_at | TNIP3 | TNFAIP3 interacting protein 3 |
| GC04P160409_at | RAPGEF2 | Rap guanine nucleotide exchange factor (GEF) 2 |
| GC02P113451_at | IL1F9 | interleukin 1 family, member 9 |
| GC10P027027_at | PDSS1 | prenyl (decaprenyl) diphosphate synthase, subunit 1 |
| GC04M139304_at | SLC7A11 | solute carrier family 7, (cationic amino acid transporter, y+ system) member 11 |
| GC04M103401_at | SLC39A8 | solute carrier family 39 (zinc transporter), member 8 |
| GC01M094066_at | GCLM | glutamate-cysteine ligase, modifier subunit |
| GC05M077816_at | LHFPL2 | lipoma HMGIC fusion partner-like 2 |
| GC17P029606_at | CCL2 | chemokine (C-C motif) ligand 2 |
| GC17P015788_at | ADORA2B | adenosine A2b receptor |
| GC22P036922_at | MAFF | v-maf musculoaponeurotic fibrosarcoma oncogene homolog F (avian) |
| GC08M029249_at | DUSP4 | dual specificity phosphatase 4 |
| GC06P151653_at | AKAP12 | A kinase (PRKA) anchor protein 12 |
| GC19M044913_at | CLC | Charcot-Leyden crystal protein |
| GC01P158975_at | SLAMF7 | SLAM family member 7 |
| GC17M035963_at | CCR7 | chemokine (C-C motif) receptor 7 |
| GC01M024044_at | FUCA1 | fucosidase, alpha-L- 1, tissue |
| GC07M149953_at | GIMAP6 | GTPase, IMAP family member 6 |
| GC01M097255_at | DPYD | dihydropyrimidine dehydrogenase |
| GC14M059132_at | RTN1 | reticulon 1 |
| GC06P088239_at | SLC35A1 | solute carrier family 35 (CMP-sialic acid transporter), member A1 |
| GC04M164668_at | MA01 | membrane-associated ring finger (C3HC4) 1 |
| GC01M016821_at | CROCCL1 | ciliary rootlet coiled-coil, rootletin-like 1 |
| GC13M047884_at | P2RY5 | purinergic receptor P2Y, G-protein coupled, 5 |
| GC13M047962_at | RCBTB2 | regulator of chromosome condensation (RCC1) and BTB (POZ) domain containing protein 2 |
| GC07M076662_at | FGL2 | fibrinogen-like 2 |
| GC11M059695_at | MS4A6A | membrane-spanning 4-domains, subfamily A, member 6A |
| GC03M152526_at | P2RY13 | purinergic receptor P2Y, G-protein coupled, 13 |
| GC01P156416_at | CD1D | CD1d molecule |
| GC08M048812_at | CEBPD | CCAAT/enhancer binding protein (C/EBP), delta |
| GC14P074815_at | FOS | v-fos FBJ murine osteosarcoma viral oncogene homolog |
