# Supplementary material for: Transcriptomic Profiling of the Development of the Inflammatory Response in Human Monocytes In Vitro
Source: PLoS One. 2014 Feb 3;9(2):e87680. doi: 10.1371/journal.pone.0087680 (PMC3912012; doi:10.1371/journal.pone.0087680)
Supplement: Table S5 — Complete list of the genes differentially expressed between untreated monocytes and M2 macrophages, extracted from database. (DOCX) [file pone.0087680.s006.docx]

**Table S5**

| Gene Id | **Symbol** | **Description** |
| --- | --- | --- |
| GC08P024297_at | ADAMDEC1 | ADAM-like, decysin 1 |
| GC03M058153_at | DNASE1L3 | deoxyribonuclease I-like 3 |
| GC12M045755_at | AMIGO2 | adhesion molecule with Ig-like domain 2 |
| GC01M160219_at | OLFML2B | olfactomedin-like 2B |
| GC01M111827_at | ADORA3 | adenosine A3 receptor |
| GC05M042835_at | SEPP1 | selenoprotein P, plasma, 1 |
| GC0XM065158_at | VSIG4 | V-set and immunoglobulin domain containing 4 |
| GC17M015073_at | PMP22 | peripheral myelin protein 22 |
| GC14M092239_at | LGMN | legumain |
| GC19P040465_at | HAMP | hepcidin antimicrobial peptide |
| GC14P092720_at | C14orf109 | chromosome 14 open reading frame 109 |
| GC06M003667_at | C6orf145 | chromosome 6 open reading frame 145 |
| GC18M019365_at | NPC1 | Niemann-Pick disease, type C1 |
| GC11M033681_at | CD59 | CD59 molecule, complement regulatory protein |
| GC05M039408_at | DAB2 | disabled homolog 2, mitogen-responsive phosphoprotein (Drosophila) |
| GC19M011546_at | ACP5 | acid phosphatase 5, tartrate resistant |
| GC07M024704_at | DFNA5 | deafness, autosomal dominant 5 |
| GC07P023252_at | GPNMB | glycoprotein (transmembrane) nmb |
| GC06M041234_at | TREM2 | triggering receptor expressed on myeloid cells 2 |
| GC12M009103_at | A2M | alpha-2-macroglobulin |
| GC01P158063_at | SLAMF8 | SLAM family member 8 |
| GC19P018358_at | GDF15 | growth differentiation factor 15 |
| GC08M082553_at | FABP4 | fatty acid binding protein 4, adipocyte |
| GC02M216516_at | MREG | melanoregulin |
| GC12M026165_at | BHLHE41 | basic helix-loop-helix family, member e41 |
| GC11P059804_at | MS4A4A | membrane-spanning 4-domains, subfamily A, member 4 |
| GC02M188039_at | TFPI | tissue factor pathway inhibitor (lipoprotein-associated coagulation inhibitor) |
| GC0XM154158_at | CLIC2 | chloride intracellular channel 2 |
| GC04M157902_at | PDGFC | platelet derived growth factor C |
| GC16M028457_at | NUPR1 | nuclear protein, transcriptional regulator, 1 |
| GC12M067531_at | CPM | carboxypeptidase M |
| GC05M101597_at | SLCO4C1 | solute carrier organic anion transporter family, member 4C1 |
| GC11P047236_at | NR1H3 | nuclear receptor subfamily 1, group H, member 3 |
| GC01M056671_at | PPAP2B | phosphatidic acid phosphatase type 2B |
| GC04P166468_at | SC4MOL | sterol-C4-methyl oxidase-like |
| GC14P060517_at | SLC38A6 | solute carrier family 38, member 6 |
| GC09P019281_at | DENND4C | DENN/MADD domain containing 4C |
| GC07M091579_at | CYP51A1 | cytochrome P450, family 51, subfamily A, polypeptide 1 |
| GC05P036642_at | SLC1A3 | solute carrier family 1 (glial high affinity glutamate transporter), member 3 |
| GC11M061323_at | FADS1 | fatty acid desaturase 1 |
| GC17P019378_at | SLC47A1 | solute carrier family 47, member 1 |
| GC14P058174_at | DACT1 | dapper, antagonist of beta-catenin, homolog 1 (Xenopus laevis) |
| GC19P050100_at | APOE | apolipoprotein E |
| GC19P050109_at | APOC1 | apolipoprotein C-I |
| GC01M055027_at | DHCR24 | 24-dehydrocholesterol reductase |
| GC02P238432_at | RAMP1 | receptor (G protein-coupled) activity modifying protein 1 |
| GC03P053855_at | IL17RB | interleukin 17 receptor B |
| GC17P031415_at | CCL18 | chemokine (C-C motif) ligand 18 (pulmonary and activation-regulated) |
| GC0XP043400_at | MAOA | monoamine oxidase A |
| GC10P102096_at | SCD | stearoyl-CoA desaturase (delta-9-desaturase) |
| GC20M043960_at | PLTP | phospholipid transfer protein |
| GC16P022732_at | HS3ST2 | heparan sulfate (glucosamine) 3-O-sulfotransferase 2 |
| GC13P097593_at | FARP1 | FERM, RhoGEF (ARHGEF) and pleckstrin domain protein 1 (chondrocyte-derived) |
| GC15P078232_at | FAH | fumarylacetoacetate hydrolase (fumarylacetoacetase) |
| GC01P022835_at | C1QA | complement component 1, q subcomponent, A chain |
| GC17P029707_at | CCL13 | chemokine (C-C motif) ligand 13 |
| GC17M031364_at | CCL23 | chemokine (C-C motif) ligand 23 |
| GC17P007883_at | ALOX15B | arachidonate 15-lipoxygenase, type B |
| GC16P029597_at | QPRT | quinolinate phosphoribosyltransferase |
| GC17M075513_at | TBC1D16 | TBC1 domain family, member 16 |
| GC01P022852_at | C1QB | complement component 1, q subcomponent, B chain |
| GC0XM037893_at | SRPX | sushi-repeat-containing protein, X-linked |
| GC11M087666_at | CTSC | cathepsin C |
| GC01M149035_at | CTSK | cathepsin K |
| GC11P086427_at | TMEM135 | transmembrane protein 135 |
| GC18M019996_at | OSBPL1A | oxysterol binding protein-like 1A |
| GC11M005203_at | HBB | hemoglobin, beta |
| GC04M084507_at | HPSE | heparanase |
| GC01P157526_at | FCER1A | Fc fragment of IgE, high affinity I, receptor for; alpha polypeptide |
| GC02M229597_at | PID1 | phosphotyrosine interaction domain containing 1 |
| GC15P037660_at | THBS1 | thrombospondin 1 |
| GC07M141273_at | CLEC5A | C-type lectin domain family 5, member A |
| GC04M075092_at | PPBP | pro-platelet basic protein (chemokine (C-X-C motif) ligand 7) |
| GC01M032573_at | MARCKSL1 | MARCKS-like 1 |
| GC04M075086_at | PF4 | platelet factor 4 |
| GC22M036290_at | LGALS2 | lectin, galactoside-binding, soluble, 2 |
| GC01P078858_at | IFI44L | interferon-induced protein 44-like |
| GC03M173706_at | TNFSF10 | tumor necrosis factor (ligand) superfamily, member 10 |
| GC11P000303_at | IFITM1 | interferon induced transmembrane protein 1 (9-27) |
| GC05P137829_at | EGR1 | early growth response 1 |
| GC12M009796_at | CD69 | CD69 molecule |
| GC01M151629_at | S100A8 | S100 calcium binding protein A8 |
| GC06M112089_at | FYN | FYN oncogene related to SRC, FGR, YES |
| GC03P144320_at | CHST2 | carbohydrate (N-acetylglucosamine-6-O) sulfotransferase 2 |
| GC04P074845_at | IL8 | interleukin 8 |
| GC04P075470_at | EREG | epiregulin |
| GC12M088244_at | DUSP6 | dual specificity phosphatase 6 |
| GC20M023008_at | CD93 | CD93 molecule |
| GC19P006838_at | EMR1 | egf-like module containing, mucin-like, hormone receptor-like 1 |
| GC14P020493_at | RNASE2 | ribonuclease, RNase A family, 2 (liver, eosinophil-derived neurotoxin) |
| GC02M156889_at | NR4A2 | nuclear receptor subfamily 4, group A, member 2 |
| GC21M043659_at | SIK1 | salt-inducible kinase 1 |
| GC19P050663_at | FOSB | FBJ murine osteosarcoma viral oncogene homolog B |
| GC16P083412_at | CRISPLD2 | cysteine-rich secretory protein LCCL domain containing 2 |
| GC01P065970_at | PDE4B | phosphodiesterase 4B, cAMP-specific (phosphodiesterase E4 dunce homolog, Drosophila) |
| GC02P069995_at | MXD1 | MAX dimerization protein 1 |
| GC05P082804_at | VCAN | versican |
| GC06M133106_at | VNN2 | vanin 2 |
| GC18P055718_at | PMAIP1 | phorbol-12-myristate-13-acetate-induced protein 1 |
| GC09M136940_at | FCN1 | ficolin (collagen/fibrinogen domain containing) 1 |
| GC01P245648_at | NLRP3 | NLR family, pyrin domain containing 3 |
| GC01M159066_at | CD244 | CD244 molecule, natural killer cell receptor 2B4 |
| GC01M167926_at | SELL | selectin L |
| GC04M084305_at | PLAC8 | placenta-specific 8 |
| GC19M010305_at | ICAM3 | intercellular adhesion molecule 3 |
| GC01M151612_at | S100A12 | S100 calcium binding protein A12 |
| GC01P191044_at | RGS2 | regulator of G-protein signaling 2, 24kDa |
